# Nature in motion: The tuning of the visual system to the spatiotemporal properties of natural scenes

**DOI:** 10.1167/jov.22.6.7

**Published:** 2022-05-19

**Authors:** Michelle M. Roberts, Mark M. Schira, Branka Spehar, Zoey J. Isherwood

**Affiliations:** 1 School of Psychology, University of Wollongong, Wollongong, NSW 2500, Australia; 2 School of Psychology, UNSW Sydney, Sydney, NSW 2052, Australia; 3 Neuroscience Research Australia, Randwick, NSW 2031, Australia; 4 Department of Psychology, University of Nevada, Reno NV 89557, USA

**Keywords:** natural scene statistics, fractals, dynamic stimuli, perceptual tuning, spatiotemporal tuning

## Abstract

Natural scenes contain several statistical regularities despite their superficially diverse appearances (e.g., mountains, rainforests, deserts). First, they exhibit a unique distribution of luminance intensities decreasing across spatial frequency, known as the 1/f^α^ amplitude spectrum (α ≈ 1). Additionally, natural scenes share consistent geometric properties, comprising similar densities of structure across multiple scales—a property classifying them as *fractal* (e.g., how the branching patterns of rivers and trees appear similar irrespective of scale). These two properties are intimately related and correlate strongly in natural scenes. However, research using thresholded noise images suggests that spatially, the human visual system is preferentially tuned to natural scene structure more so than 1/f^α^ spectra. It is currently unclear whether this dependency on natural geometry extends to the temporal domain. We used a psychophysics task to measure discrimination sensitivity toward two types of synthetic noise movies: gray scale and thresholded (*N* = 60). Each movie type shared the same geometric properties (measured fractal *D*), but substantially differing spectral properties (measured α). In both space and time, we observe a characteristic dependency on stimulus structure across movie types, with sensitivity peaking for stimuli with natural geometry despite having altered 1/f^α^ spectra. Although only measured behaviorally, our findings may imply that the neural processes underlying this tuning have developed to be sensitive to the most stable signal in our natural environment—*structure* (e.g., the structural properties of a tree are consistent from morning to night despite illumination changes across time points).

## Introduction

Our visual experience of the natural world does not occur in stasis, but extends dynamically over space and time. Given this perceptual complexity, is the human visual system preferentially tuned to specific properties within dynamic scenes? Of relevance to this question are several statistical regularities across natural scenes: first, the amplitude of intensity (luminance) variations decreasing as a function of spatial frequency. This power-law relationship is known as the 1/f^α^ amplitude spectrum and holds across natural images despite their varied appearances (Burton & Moorhead, 1987; Field, 1987). When plotted on a log–log axis, the slope (α) of the line typically ranges from 0.8 to 1.6, with an average value of approximately 1.2 (Field, 1987; Tolhurst et al., 1992; Field & Brady, 1997; Webster & Miyahara, 1997; Oliva & Torralba, 2001; Hansen & Essock, 2005; Hansen & Hess, 2006; Flitcroft et al., 2020).

Preferential tuning to this luminance-based property has been established both psychophysically and neurophysiologically. Optimal visual sensitivity has been consistently observed toward stimuli exhibiting 1/f^α^ amplitude spectral characteristics within the intermediate range (α ∼1.2) (Knill et al., 1990; Field & Brady, 1997; Tolhurst & Tadmor, 2000; Párraga et al., 2000; Billock et al., 2001b; Hansen & Hess, 2006; Ellemberg et al., 2012; Spehar et al., 2015). Visually, these findings typically reveal non-linear tuning profiles resembling an inverted “U” shape, with sensitivity peaking for natural slopes and falling off as spectral statistics depart this intermediate range. From a neurobiological perspective, it has been theorized that this tuning may result from human visual and perceptual systems adapting to ecological constraints throughout their developmental history (Simoncelli & Olshausen, 2001; Geisler, 2008).

Despite the robust association between heightened visual sensitivity and natural 1/f^α^ spectra, a degree of ambiguity remains as to whether the spectral properties are predominantly driving this response. Because naturalistic gray scale stimuli contain full-spectrum luminance information, multiple implicit edges exist wherever contrasting luminance values are densely packed within the stimulus. In this way, naturalistic stimuli contain inherent geometry that accompanies the 1/f^α^ spectral distribution. In a typical natural scene, the relative amount of high and low spatiotemporal frequencies is unchanged when the viewing angle is altered, that is, any section of the 1/f^α^ amplitude–frequency relationship resembles the whole (Ruderman, 1994; Olshausen & Field, 2000; De Cesarei et al., 2017). This self-similarity across multiple spatial and temporal scales classifies natural scene spectra as *fractal,* a property that can be captured by a parameter known as fractal dimension (*D*) (Ruderman & Bialek, 1994; Ruderman, 1997). The fractal dimension (*D*) is computed by binarizing an image and quantifying the amount of fine spatial detail at boundary edges between the filled and empty regions (Cutting & Garvin, 1987; Feder, 2013). Spatial *D* values range from 1 to 2, and reflect the ratio of coarse-to-fine structure in a pattern; *D* values approaching 2 signify a greater degree of intricate spatial detail. As such, *D* values also indicate the visual complexity of a pattern, with higher *D* values indicating greater structural complexity (Mandelbrot et al., 1983). In the temporal domain, depending on the measuring method, fractal *D* can also range from 1 to 2 and refers to the degree of self-similar information in a scene as measured over multiple time points (Cutting et al., 2018). This statistical self-similarity is described as fractal self-affinity within the time series (Pilgrim & Taylor, 2019). A natural scene exhibiting 1/f^α^ properties therefore also contains embedded, and distinctly measurable, fractal geometry (Soille and Rivest, 1996; Spehar et al., 2003).

Historically, investigations of visual sensitivity toward naturalistic noise involve manipulating 1/f^α^ spectrum information across a suite of stimuli, with sensitivity consistently peaking in response to intermediate 1/f^α^ spectral properties (α ≈ 1). However, these findings fail to consider any influence of the implicit fractal geometry of the stimuli. To disambiguate any discrete influences of 1/f^α^ spectra and natural fractal structure on visual sensitivity, Isherwood et al. (2017) used both gray scale and thresholded noise stimuli. The authors generated a set of gray scale stimuli ranging in spatial slope (α) from 0.25 to 2.25 (in steps of 0.50). From these, thresholded stimuli were produced by binarizing, at mean luminance, the original gray scale stimuli. This procedure decreases the luminance-based information in the stimulus, exposing only the edges where high-contrast luminance variations are densely packed. The subsequent thresholded stimulus retains the same scaling properties as the gray scale original; in this way, thresholding makes the implicit geometry of a gray scale stimulus explicit. After this process, measurements between the gray scale and thresholded stimuli yielded an identical fractal dimension (*D*), but notably shallower spectral slope (α) values. Despite these changes to the spectral properties of the stimuli, cortical activation remained statistically equal in response to both image types. Isherwood et al. (2017) concluded that sensitivity responses in the early visual cortex seem to be driven primarily by the structural characteristics of the stimuli; the sensitivity was unaffected by changes to the luminance-based properties. It is currently unknown whether the critical role of scene structure extends to sensitivity responses in the temporal domain.

### The 1/f^α^ amplitude spectrum in the temporal domain

Research investigating the temporal regularities of natural scenes initially applied correlational analyses to movies of natural scenes (Dong & Atick, 1995). It was observed that the luminance intensities of spatially adjacent pixels were strongly correlated across the time-varying sequence. In contrast, fluctuations across time in a pixel's luminance had little to no similarity to the luminance of pixels located further away. In light of these temporal correlations, several researchers have used Fourier analyses to examine amplitude across *temporal* frequency. Because natural scenes are composed of spectral energy distributed across both high and low temporal frequencies, studies using this method have found that dynamic natural scenes display commensurate 1/f^α^ behavior. Analysis of natural time series (e.g., movies, documentaries) reveals temporal slope values analogous to natural *spatial* spectra, with natural scenes displaying an average temporal slope of approximately 1 (Eckert & Buchsbaum, 1993; Van Hateren, 1993, 1997; Billock et al., 2001b; Bex et al., 2005).

Following the findings of Dong and Atick (1995), few studies have investigated visual sensitivity to 1/f^α^ spectra across various temporal manipulations (Billock et al., 2001a; Baker & Graf, 2009; Cass et al., 2009). Billock et al. (2001a) examined discrimination toward dynamic filtered noise stimuli varying across a wide range of spatiotemporal slopes. The use of computationally generated filtered noise allows precise control of the 1/f^α^ spectral characteristics of the stimuli while also minimizing the confounding influence of measuring perceptual processes related to object recognition, memory, or emotion (Willenbockel et al., 2010). Temporal just-noticeable-difference thresholds were measured via an adaptive staircase procedure, with the spatial exponent held constant for each block (spatial slope = 0.4–2.2, step size 0.2) as the temporal slope adaptively varied within blocks (temporal slope = 0.2–1.4, step size 0.2). Irrespective of spatial slope, peak temporal discrimination was measured toward stimuli when the temporal slope was between 0.8 and 1.0; that is, toward the mid-range slope values prevalent in dynamic natural scene spectra (Billock et al., 2001a).

These findings were supported by those of Isherwood et al. (2021) using a psychophysics task to investigate spatiotemporal sensitivity toward a wider range of 1/f^α^ spectra. Synthetic gray scale stimuli were generated at the spatial slope conditions of 0.25, 1.25, and 2.25. These stimuli were then manipulated dynamically to correspond with five different temporal slope conditions ranging from 0.25 to 2.25, increasing in step sizes of 0.50. Participants were tasked with discriminating a target stimulus moving at a different speed relative to three distractor stimuli in a four-alternative forced choice odd-one-out task. Discrimination sensitivity was highest in response to stimuli with the most natural spatiotemporal spectra, with the peak sensitivity (1/threshold) observed for stimuli with a spatial slope and a temporal slope of 1.25. In agreement with Billock et al. (2001a), these findings show that dynamic visual discrimination is easiest when stimuli most closely approximate the spatiotemporal properties of natural scenes.

### The present study

Natural scenes, and our visual experience of them, exist dynamically in both space and time. Because the spectral energy within dynamic natural scenes is distributed across both high and low temporal frequencies, an amplitude-temporal frequency relationship has been identified that is essentially identical to the 1/f^α^ power-law observed in static natural scenes. The present study aims to extend the findings of Isherwood et al. (2021) by examining perceptual sensitivity in response to gray scale and thresholded filtered noise movies. The addition of a dynamic thresholded movie type permits an exploration of visual sensitivity toward spatiotemporal fractal properties. We measure discrimination sensitivity across two distinct sets of dynamic stimuli with widely varying spectral characteristics, yet highly similar fractal characteristics. Given past findings in the spatial domain (Isherwood et al., 2017), we predict that optimal performance in our discrimination task (four-alternative forced choice) will be observed in response to stimuli with the most natural fractal geometry in both space and time (α = 1.25), irrespective of the movie type. Additionally, we predict that tuning curves will follow an inverted U profile for each movie type (peaking for the most natural stimulus in the set), replicating the typical response pattern of previous natural scene sensitivity research.

## Materials and methods

### Design

The present study used a 2 (Movie type) × 3 (Spatial slope) × 5 (Temporal slope) mixed model design. Each level of the between-subjects factor of movie type (gray scale, thresholded) contained the within-subjects repeated measures factors of the spatial slope (0.25, 1.25, and 2.25) and the temporal slope (0.25, 0.75, 1.25, 1.25, and 2.25). Participants were pseudo-randomly allocated to either the gray scale or thresholded movie type.

### Participants

Based on the effect sizes reported by Isherwood et al. (2021), power analyses conducted using G*Power confirmed that a sample size of 60 participants (30 per movie type) yielded greater than 95% power to detect between-subjects effects (Erdfelder et al., 1996). All 60 participants had normal or corrected-to-normal vision and were naive to the purposes of the experiment. The study was undertaken with the written consent of all participants. Ethics approval was provided by the Human Research and Ethics Committee at the University of Wollongong, Australia (Approval Number: 2019/193).

### Apparatus

The stimuli were presented on a 27-inch DELL (U2718Q) LCD monitor. The screen resolution was set to 3840 × 2160 at a refresh rate of 60 Hertz. An adjustable chin and forehead rest was used to center participants’ head position, fixed at a viewing distance of 50 cm. Owing to computational limitations in both memory and graphics processing, the most suitable stimulus size was 256 × 256 pixels across 128 frames, subtending a visual angle of 5.10°. The experiment was conducted in a dark cubicle with button-press responses collected using a regular computer keyboard.

### Stimuli

**Figure 1.**
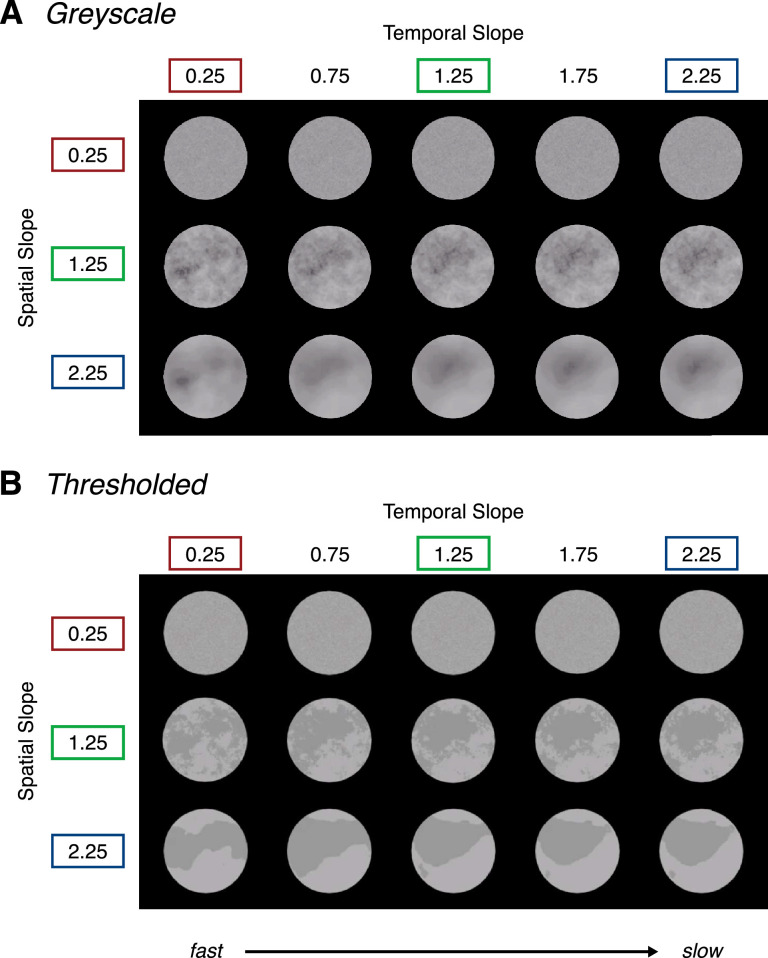
Gray scale stimuli (A) and thresholded stimuli (B) manipulated across three spatial slope conditions (0.25, 1.25, and 2.25) and five temporal slope conditions (0.25, 0.75, 1.25, 1.75, and 2.25) at 6% RMS contrast. Note that the contrast of the stimuli has been increased for visibility in print. To view the experimental stimuli in motion, see the following Movies: (1) gray scale: https://osf.io/8ehpc/ (2) thresholded: https://osf.io/vzh92/.

Stimuli were generated and presented in MATLAB (version 9.5, R2018b) using the Psychophysics Toolbox (Pelli, 1997; Brainard, 2019). The stimuli varied across two different movie types: gray scale and thresholded (Figure 1). Each stimulus type was generated via a sequential process which alters their photometric properties across each iteration, while preserving the geometric, fractal-like characteristics across each set of stimuli.

#### Gray scale stimuli

Synthetic gray scale movies were generated the same way as in Isherwood et al. (2021). Gray scale movies were first generated from a seed of random noise with randomly distributed pixel intensities. A custom MATLAB code was used to transform the distribution of pixel intensities to the requisite 1/f^α^ slopes (*make_fractal_3D.m*: https://osf.io/w5tvn/). To achieve this, an FFT was used to break the noise seed down to its component sine and cosine waves. The component spatial frequencies and temporal frequencies were adjusted to correspond with an amplitude spectrum conforming to 1/f^α^ in space and time at 30% root mean square (RMS) contrast with a mean luminance of 0.5 and a standard deviation of 0.15. The resulting stimuli varied in their spectral distributions across three spatial slope conditions (α = 0.25, 1.25, 2and .25). Each of these spectral distributions were dynamically manipulated across five temporal slope conditions (α = 0.25, 0.75, 1.25, 1.75, and 2.25). This process resulted in 15 unique gray scale spatiotemporal slope combinations. The stimulus size was 256 × 256 pixels across 128 frames (presented at 60 frames/second), subtending a visual angle of 5.10°.

#### Thresholded stimuli

**Figure 2.**
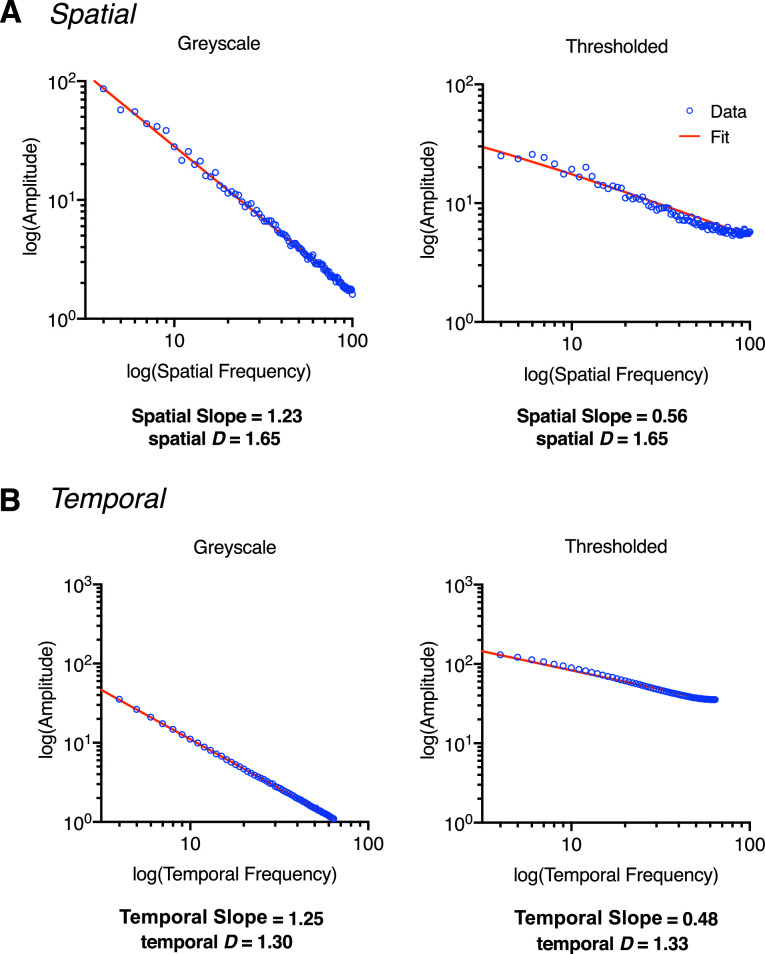
Stimulus generation of a gray scale and thresholded stimuli, generated here with an input spatial slope of 1.25 and an input temporal slope of 1.25. (A) Plots for the measured spatial slope (α) and measured spatial D values for a gray scale stimulus (left) and thresholded stimulus (right). (B) Plots depicting the measured temporal slope (α) and measured temporal D values for a gray scale stimulus (left) and thresholded stimulus (right). Note the shallower slope values (spatial slope and temporal slope) for thresholded stimuli following the thresholding process. Despite this change, fractal D values remain highly similar both spatially and temporally. Note that the amplitude spectra plotted in (A) are from a representative frame: 64/128. The reported spatial slope is an average across frames.

To generate the thresholded movie type, each frame of the gray scale stimulus set was thresholded at the mean luminance. Typically, this manipulation results in the remeasured (output) spatial and temporal slopes of the stimuli being shallower than those of the gray scale condition, primarily owing to the altered photometric information within the stimulus (Spehar et al, 2016; Isherwood et al., 2017) Despite these changes in measured output slope, the underlying fractal properties of the thresholded stimuli remain highly similar to their gray scale counterparts (Figure 2).

### Contrast control

We also equated the RMS contrast of our stimuli. The importance of contrast normalization when examining sensitivity across differing spatial frequencies is critical for making accurate conclusions regarding perceptual responses (Campbell & Robson, 1968; Perfetto, Wilder & Walther, 2020). The Spectrum, Histogram, and Intensity Normalization and Equalization toolbox (Willenbockel et al., 2010) was used to match RMS contrast across all experimental stimuli at 6%, with a mean luminance of 0.5 and a standard deviation of 0.03 within MATLAB. A higher contrast level was not used owing to the difficulty in matching RMS contrast across the two movie types. Movies of the contrast controlled stimuli are viewable here: 1) gray scale, https://osf.io/8ehpc/; 2) and thresholded, https://osf.io/vzh92/.

### Image analysis

#### The 1/f^α^ amplitude spectrum

To measure the output spatial slope of the stimuli, within MATLAB, the 1/f^α^ spectrum of each stimulus was plotted and fitted for each frame (128) separately in linear space before being transformed to a log–log axis. To measure the output temporal slope of the stimuli, this process involved averaging the spatial luminance intensity of each frame across x and y dimensions (256 × 256) then plotting the temporal amplitude spectrum (Z dimension) across 128 frames and fitting the data in linear space. After this step, the linear fit of the data was replotted in log–log space. Transformation to 8-bit causes a loss of information owing to converting these values to the nearest integer, which is emphasized when plotting and fitting data on a log–log axis. This variability owing to 8-bit conversion rounding errors is observed in both the spatial and temporal domains for both gray scale and thresholded movie types. For simplicity, here we report the measured spatial slope and temporal slope before 8-bit conversion. Refer to Figure S1, Figure S2, and Table S1 and Table S2 in the Supplementary Materials for measured slope values after 8-bit conversion.

For the thresholded movie type, the thresholding procedure also changes the output slope since binarizing consequentially increases energy across high spatial and temporal frequencies. These effects are particularly evident for spatial slope 2.25, temporal slope 2.25. Despite this, there is notably less variability in fractal *D* values between movie types—see Table 1 (spatial) and Table 2 (temporal) for values, and Figure 5 (spatial) and Figure 6 (temporal) for comparison. See Table S5 (spatial) and Table S6 (temporal) in the Supplementary Materials for a more detailed summary of slope and fractal D estimates. Terminology referring to spatial slope and temporal slope will refer to the input α value unless otherwise stated.

**Table 1.** Spatial measurements: Measured 1/f^α^ slope (spatial slope) and spatial fractal D values across gray scale and thresholded movie types in the spatial domain

	Movie type			Measured difference
	Spatial slope (input)	Gray scale	Thresholded	Gray scale and thresholded
Spatial slope (measured)	0.25	0.24 (<0.01)	0.02 (<0.01)	0.06
	1.25	1.23 (0.01)	0.56 (<0.01)	0.17
	2.25	2.22 (0.11)	1.10 (0.01)	0.85
Spatial fractal *D* (measured)	0.25	1.97 (<0.01)	1.97 (<0.01)	0.00
	1.25	1.65 (0.01)	1.65 (0.01)	0.00
	2.25	1.00 (0.02)	0.99 (0.02)	0.01

Notes: The reported spatial slope values have been averaged across the 640 frames presented in each **t**emporal **s**lope condition (128 frames x 5 **t**emporal **s**lope). Values in parentheses indicate standard deviation. Results have been rounded to two decimal places.

**Table 2.** Temporal measurements: Measured temporal slope and temporal fractal D values across gray scale and thresholded movie types in the temporal domain

	Movie type			Measured difference
	Temporal slope (input)	Gray scale	Thresholded	Gray scale and thresholded
Temporal slope (measured)	0.25	0.25 (0.01)	0.08 (0.08)	0.19
	0.75	0.75 (0.02)	0.21 (0.22)	0.53
	1.25	1.25 (0.01)	0.42 (0.32)	0.82
	1.75	1.75 (<0.01)	0.57 (0.35)	1.18
	2.25	2.26 (0.03)	0.61 (0.35)	1.65
Temporal fractal *D* (measured)	0.25	1.69 (0.01)	1.79 (0.02)	0.10
	0.75	1.50 (<0.01)	1.58 (0.01)	0.07
	1.25	1.29 (0.01)	1.33 (<0.01)	0.03
	1.75	1.19 (0.01)	1.22 (0.02)	0.02
	2.25	1.17 (<0.01)	1.19 (0.02)	0.02

Notes: The reported temporal slope values have been averaged across the three **s**patial **s**lope conditions (0.25, 1.25, and 2.25). Values in parentheses indicate standard deviation. Results have been rounded to two decimal places.

The MATLAB code used to measure the 1/f^α^ spatiotemporal amplitude spectrum of our stimuli were slightly modified versions of the scripts used in Isherwood et al. (2021), 1) spatial spectrum (*calc_spatialSlope_R2_fit.m*), https://osf.io/2sfmh/, and 2) temporal spectrum (*calc_temporalSlope_R2_fit.m*), https://osf.io/qvfud/. In the original MATLAB code from Isherwood et al. (2021), an Akaike information criterion was used to determine how many points should (or should not) be removed to best fit the amplitude spectrum in linear space. Points were removed owing to systematic deviations from linearity at lower frequencies which would greatly affect the fit. Model comparisons were conducted by comparing a linear fit of the amplitude spectrum in log–log space versus a quadratic fit in linear space. The point at which both models performed equally indicated the number of points that should be removed. For the set of stimuli used in the present study, this method yielded poor fits of the amplitude spectrum (see Figure S3). To account for this, we instead used a criterion where we only fit the data in linear space with a quadratic function with and without the bottom 2% of points. We then compare all the fits; the fit with the highest R2 was selected as the final fit and estimate of the amplitude spectrum slope.

#### Fractal dimension

**Figure 3.**
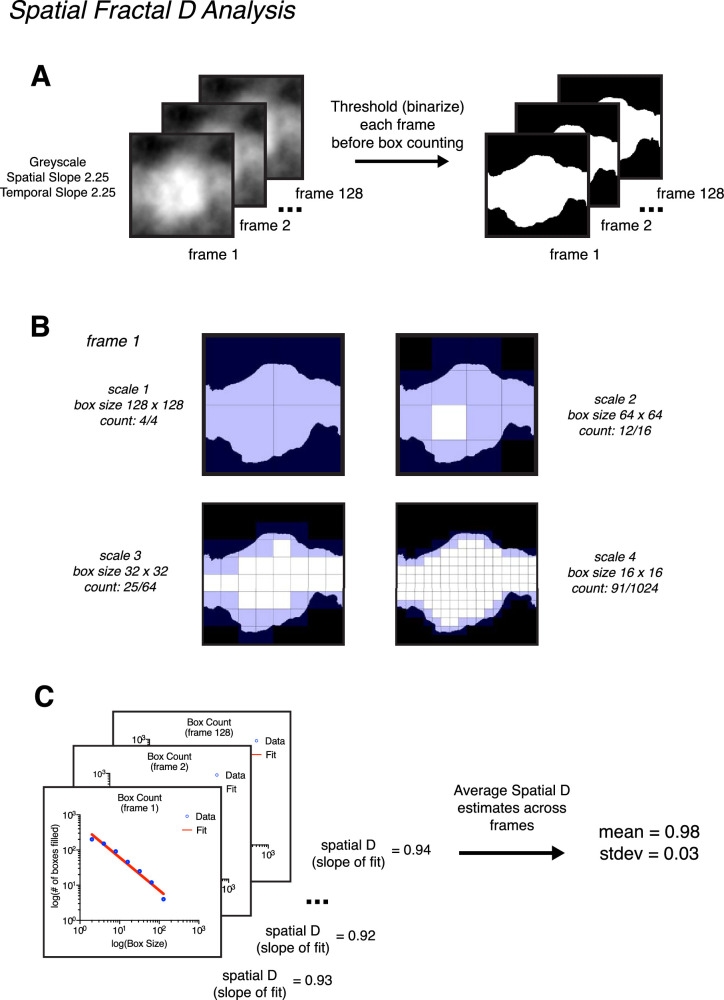
Demonstration of how spatial fractal D is calculated using stimulus condition gray scale spatial slope of 2.25 and temporal slope of 2.25. (A) Before box counting, the frames of each movie stimulus were thresholded (i.e., binarized). This consisted of converting all pixels above mean luminance to white (255) and all pixels below to black (0). B) The box-counting procedure consisted of overlaying each frame with a mesh grid of equally sized boxes. Boxes which contained pixel values 0 and 1 were counted as occupied. This procedure was conducted across multiple spatial scales, and the maximum number of scales analyzed was equal to maximum number of times the image size could be divided by 2, with the smallest box size being 2. For stimuli in the present study, seven scales were counted (256 -> 128, 64, 32, 16, 8, 4, 2). Only four scales are depicted for brevity. (C) After box counting, spatial fractal D is calculated by fitting the slope of the relationship between box size and box occupancy a log–log axis. This value was calculated for each frame separately. The final spatial reported value is the average across all frames.

Spatial fractal *D* was measured using a box-counting technique on each individual frame of our stimuli (Bies et al., 2016; Viengkham & Spehar, 2018; Viengkham et al., 2019). This technique consists of first thresholding an image at mean luminance (Figure 3A), then dividing the thresholded image into a grid of equally sized squares (boxes) to count how many are occupied by the pattern of the image (Figure 3B). This process is repeated using smaller and smaller box sizes, which capture finer details within the image. For natural scenes, the relationship between box size and occupation follows a power law. When plotted on a log–log axis, the slope of this relationship corresponds with the spatial fractal *D* of an image (Figure 3C). For each stimulus, spatial fractal *D* values were measured for each frame separately. We report the average spatial fractal *D* value across frames and the standard deviation. The MATLAB script used to measure spatial fractal *D* was *measure_fractalD_spatial.m*, which is available on OSF here: https://osf.io/9fnrg/.

**Figure 4.**
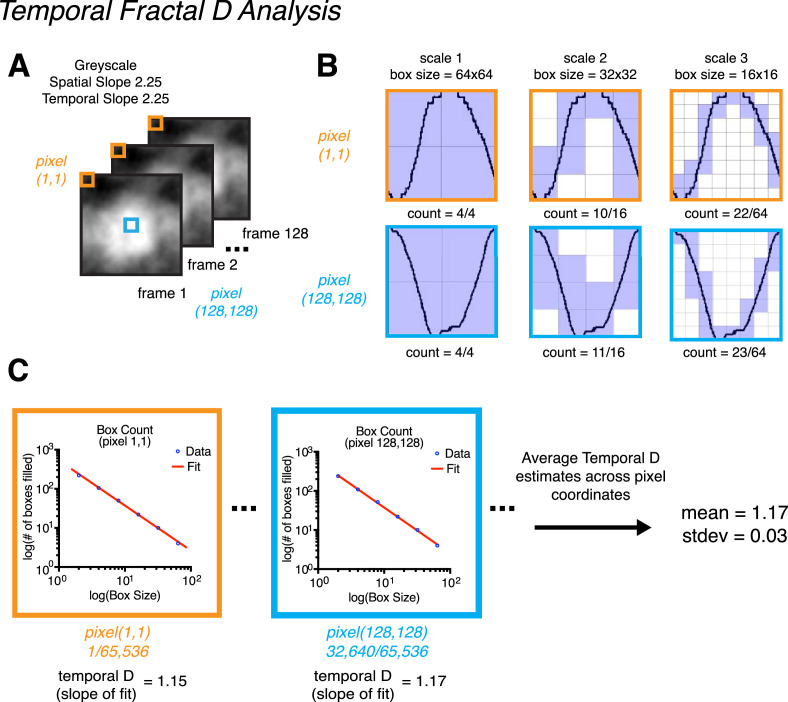
Demonstration of how temporal fractal D is calculated using stimulus condition gray scale spatial slope of 2.25 and a temporal slope of 2.25. (A) Depiction of an example stimulus, and two example pixel coordinates across frames: pixel (1, 1) in orange and pixel (256, 256) in blue. The temporal fractal D values we report were calculated by averaging across temporal fractal D estimates made for each pixel coordinate within each stimulus. Specifically, for each pixel coordinate (256 × 256 = 65,536 coordinates total) we plotted luminance as a function of time (frames) and conducted a box-counting procedure on each plot. (B) The box-counting procedure consisted of overlaying each plot with a mesh grid of equally sized boxes. As in our spatial fractal D procedure, boxes that contained pixel values 0 and 1 were counted as occupied. This procedure was conducted across multiple spatial scales, and the maximum number of scales analyzed was equal to maximum number of times the image size could be divided by 2, with the smallest box size being 2. For stimuli in the present study, six scales were counted (128 frames -> 64, 32, 16, 8, 4, 2). Only three scales for pixel (1, 1) and pixel (128, 128) are depicted for brevity. (C) After box counting, temporal fractal D was calculated for each pixel coordinate by fitting the slope of the relationship between box size and box occupancy a log–log axis. The final reported temporal fractal D value is the average across all pixel coordinates.

The temporal fractal *D* was measured in essentially the same way as spatial fractal *D*. However, box counting was instead conducted on luminance changes across movie frames (Z) in time rather than across spatial dimensions (X and Y). To do this, for each pixel in the movie (X, Y) the luminance was plotted across frames as a one-dimensional (1D) plot (see Figure 4^5^,^6^A). Box counting was conducted on each 1D plot in the same way as the spatial domain, where a grid of boxes across different scales (large to small) was overlayed on the plot to count how many boxes were occupied by the trace in the 1D plot (see Figure 4B). We then plotted box size as a function of box occupancy on a log–log axis and the slope of this relationship we refer to as the temporal fractal *D* (Figure 4C). We report the average temporal fractal *D* value across pixels and the standard deviation. Unlike in the spatial domain, for the purposes of box counting we did not need to threshold the 1D plot as the temporal data is already binary. The MATLAB script used to measure temporal fractal *D* was *measure_fractalD_temporal.m*, which is available on OSF here: https://osf.io/nt24p/. Note that, owing to the low RMS contrast level used in the present study (6%), we opted to measure the temporal fractal *D* across pixels individually rather than across a spatially collapsed average. For particular stimulus conditions (e.g., a gray scale spatial slope of 2.25 and a temporal slope of 2.25), the luminance change over time was minimal and of low magnitude (pixel values often being between 127.0 and 127.5 across frames). When collapsing across spatial dimensions for this particular sort of stimulus, plotting luminance across frames with the DC component removed artificially inflates the temporal fractal *D* measurement (see Figure S4 in the Supplementary Materials). For our gray scale spatial slope of 2.25 and temporal slope of 2.25 stimulus, averaging across the X and Y dimensions yields a temporal fractal *D* measurement of 1.77 versus 1.17 when measured without collapsing across these dimensions. As such, to avoid this issue, we opted to measure temporal fractal *D* across individual pixels.

**Figure 5.**
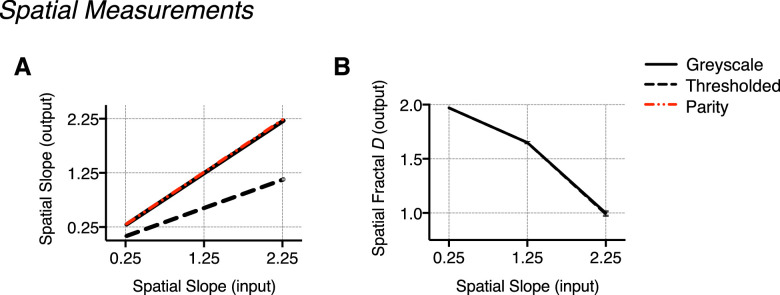
Measurements in the spatial domain: Measured output 1/f^α^ slope and fractal D values. (A) Output spatial slope plotted as a function of the input spatial slope and displayed against a line of parity (indicating identical input/output values). Averaged across temporal slope conditions, output spatial slope conditions can be seen to deviate markedly between movie types, particularly at a spatial slope of 2.25. (B) Spatial fractal D output measured for each movie type plotted as a function of spatial slope. (C) There is comparatively minimal variance in fractal D compared with the range of measured output for α, particularly at a spatial slope of 2.25. This illustrates the high degree of structural similarity retained despite the vast photometric changes to the stimuli. Error bars indicate the standard error of the mean (no visible error bars indicate the variance to be smaller than the physical size of the data point on the plot).

**Figure 6.**
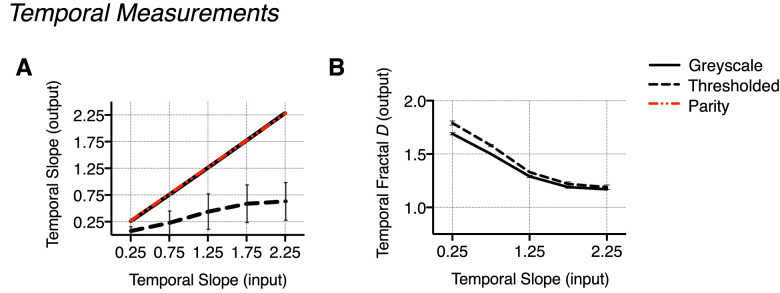
Measurements in the temporal domain: output 1/f^α^ slope and fractal D values. (A) Output temporal slope averaged across spatial slope, plotted as a function of input temporal slope and displayed against a line of parity (no difference). Similar to the measured output slopes in the spatial domain, output temporal slope conditions are notably different between movie types. (B) Output temporal fractal D values averaged across the spatial slope for each movie type and plotted as a function of temporal slope. (C) Plotting the measured difference for each parameter reveals substantially less difference in measured D between movie types when compared to the range of the difference in measured α, which is particularly large at the higher temporal slope conditions. Error bars indicate the standard error of the mean (no visible error bars indicate the variance to be smaller than the physical size of the data point on the plot).

### Visual sensitivity measurements

A four-alternative forced choice odd one out paradigm was used to measure participants’ just noticeable difference thresholds. The Bayesian adaptive psi procedure was used to determine the discrimination threshold contrast necessary for a participant to detect increases and decreases in input 1/f^α^ temporal slope (Kontsevich & Tyler, 1999). Each trial consisted of four dynamic stimuli simultaneously presented in quadrant formation on a mean luminance screen. Each formation contained three distractor stimuli moving at an identical rate (i.e., the same temporal slope) and one target stimulus moving at a different rate (i.e., appearing to either have faster or slower luminance modulation dependent on a decrease or increase in temporal slope). Participants were required to indicate which stimulus was moving at a different rate relative to the others (the odd one out) via button press.

Stimuli were presented in a block design, with the combination of three spatial slope conditions and 5 temporal slope conditions yielding 15 blocks in total. The order of block presentation was randomized for each participant. Within each block, all stimuli were displayed with the same SS and a base temporal slope that either increased or decreased based on participant responses. Trial-to-trial changes in the temporal slope of the target stimulus were adaptively determined by a psi staircase design across 30 ascending and 30 descending intermixed trials within the run, set to estimate the sensitivity threshold of the observer at a 75% correct response rate. Across 15 blocks, with 60 trials each, participants completed 900 trials in total. Including optional breaks between blocks, the average time to complete the experiment was approximately 1 hour.

Each trial in the experimental task began with a central fixation point (4 × 4 pixels, 0.08° visual angle) presented for approximately one second followed by four experimental stimuli displayed for approximately 2.13 seconds. Participant response time was unlimited. Trial-specific auditory feedback was provided in real time, whereby one tone indicated a correct response and two tones indicated an incorrect response.

Stimulus presentation was randomized within each quadrant, ensuring an equal probability of target stimulus location in each trial. Additionally, the stimuli were rotated relative to each other across the four quadrants and each stimulus was presented with contrast jittered between 70%, 80%, 90%, and 100% of its original value. This was done to prevent learning effects or the use of image matching strategies based on factors other than temporal slope; these techniques have been found to influence sensitivity scores (Fahle & Edelman, 1993; Herzog & Fahle, 1997).

### Procedure

Before undertaking the experimental task, participants were verbally briefed on the task requirements and instructed to adjust the chin rest to a comfortable position. Participants then undertook 15 practice trials in the presence of the experimenter to ensure task competency. After this, participants commenced the experimental task without the presence of the experimenter in the testing cubicle.

### Data processing

Initial analyses were conducted using custom MATLAB scripts. Discrimination thresholds were averaged across up and down staircases. The raw de-identified data files for each movie type are available at 1) gray scale condition, https://osf.io/hm2g6/ and 2) thresholded condition, https://osf.io/tbdzc/. Discrimination threshold values were inverted to give a final index of discrimination sensitivity (1/threshold) and analyzed using IBM SPSS statistics software (version 24). This involved fitting a repeated-measures general linear model to the data to perform a mixed-model analysis of variance. Degrees of freedom for all reported statistics were corrected using Greenhouse–Geisser estimates of sphericity (ε = 0.348 and ε = 0.403, respectively).

## Results

**Figure 7.**
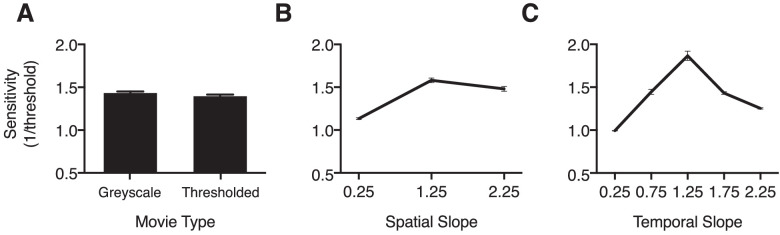
Sensitivity across movie type and spatiotemporal conditions. (A) Main effect of movie type. Sensitivity averaged across spatial slope and temporal slope conditions and plotted as a function of movie type. Overall sensitivity was similar across both movie type conditions. (B) Main effect of SS. Sensitivity averaged across movie type and temporal slope, plotted as a function of spatial slope. Peak sensitivity can be observed in response to the most natural spatial slope in the set—spatial slope 1.25. (C) Main effect of the temporal slope. The overall temporal tuning profile follows an inverted-U curve, with maximal sensitivity observed for the most natural temporal slope in the set (1.25). Notes: Error bars indicate the standard error of the mean for each data point (no visible error bars indicate the variance to be smaller than the physical size of the data point on the plot). All pairwise comparison data are included in the Supplemental Materials in Table S7 (spatial slope) and Table S8 (temporal slope).

To evaluate our hypothesis—that visual sensitivity is modulated by the underlying fractal geometry of a dynamic stimulus, as opposed to its spectral properties—we conducted a 2 × 3 × 5 repeated-measures analysis of variance with sensitivity (1/threshold) as the dependent variable, movie type (gray scale, thresholded) as a between-subjects independent variable, and two within-subjects independent variables of spatial slope (0.25, 1.25, and 2.25) and temporal slope (0.25, 0.75, 1.25, 1.75, and 2.25). The omnibus analysis of variance revealed no significant main effect of movie type, *F* (1, 58) = 1.01, *P* = .318, η_p_^2^ = 0.017, indicating that observers in the thresholded condition did not significantly differ in sensitivity performance to those in the gray scale condition (Figure 7A). The main effect of SS was found to be statistically significant, *F* (2, 114) = 168.36, *P* < .001, η_p_^2^ = 0.744, with Bonferroni-corrected post hoc comparisons indicating that discrimination sensitivity was highest in response to the intermediate SS of 1.25 (*P* < .001) (Figure 7B). Additionally***,*** the main effect of temporal slope was statistically significant, *F* (2, 89) = 164.94, *P* < .001, η_p_^2^ = .740, with observers’ sensitivity significantly higher for the temporal slope value of 1.25, *P* < .001 (Figure 7C). This finding was observed regardless of the spatial characteristics of the stimuli, indicating that sensitivity was optimal in response to stimuli exhibiting the most natural statistical motion—regardless of the level of detail within the stimulus.

### Interactions

**Figure 8.**
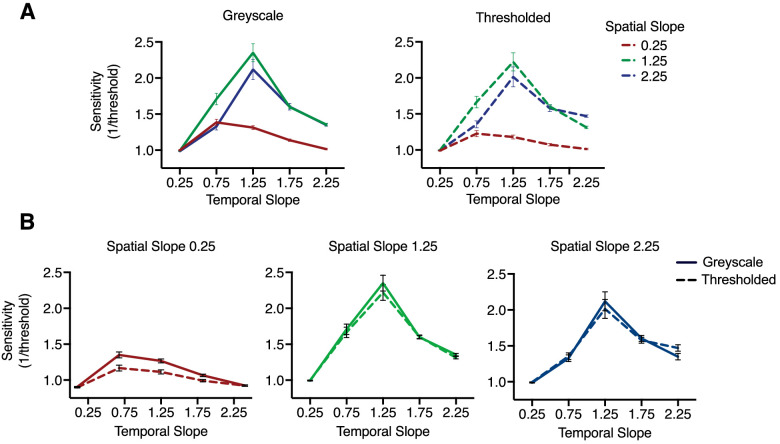
Sensitivity for each movie type (top) and discrete spatial slope conditions (below). (A) Gray scale (left) and thresholded (right) tuning curves plotted for each spatial slope as a function of temporal slope. The pattern of the response profiles is highly similar for each movie type. (B) Sensitivity toward each spatial slope discretely (from left to right = 0.25, 1.25, 2.25) plotted as a function of the temporal slope. Despite an overall decreased sensitivity toward the spatial slope 0.25, the tuning curves for each spatial slope remain similar, with each peaking for intermediate range spatial slope and temporal slope values. A subtle shift in peak can be seen when the stimuli concur in space and time (e.g., a spatial slope of 1.25 and a temporal slope of 1.25). This shift in peak is less clear toward a spatial slope of 2.25, where the peak sensitivity is observed toward the natural temporal slope of 1.25; however, sensitivity does not drop off, but remains slightly elevated at the steeper temporal slope conditions of 1.75 and 2.25. Error bars indicate standard error of the mean.

There was a significant spatial slope by temporal slope interaction, *F* (3, 187) = 40.96, *P* < .001, η_p_^2^ = .414, whereby increased sensitivity emerged when stimuli concurred in space and time. Although the overall sensitivity was highest toward stimuli with the most natural spatial and temporal characteristics (spatial slope of 1.25, temporal slope of 1.25), peak sensitivity was observed to shift leftward for a spatial slope of 0.25 for shallower temporal slope conditions (0.25 and 0.75). Peak sensitivity toward a spatial slope of 2.25 was evident at the most natural temporal slope (1.25); however, sensitivity to this spatial slope did not decrease, but remained elevated between temporal slope conditions of 1.75 and 2.25. The nature of this interaction was apparent irrespective of movie type, with highly similar tuning profiles evident for each spatial slope. Figure 8A displays the tuning curves for each movie type separately (top row), as well as individual plots of the tuning curves for each movie type depicted as a function of temporal slope (Figure 8B).

## Discussion

It is well-established that the human visual system is optimally tuned to process natural 1/f^α^ spectra. The present study investigated whether the visual system is spatiotemporally tuned to natural fractal geometry more so than the 1/f^α^ photometric characteristics of a stimulus. We hypothesized that, if the visual system is tuned to the photometric characteristics of dynamic natural scenes, discrimination sensitivity would differ between movie types owing to the altered measured 1/f^α^ spectra of thresholded stimuli in comparison with their gray scale counterparts. However, if the visual system is preferentially tuned to the geometry within a dynamic scene, performance should remain equivalent between movie types despite these changes to the 1/f^α^ spectra of the stimuli.

In support of our predictions, peak sensitivity was observed in equal magnitude toward gray scale and thresholded stimuli with the most natural spatial and temporal structure. Spatially, these findings agree with those of Isherwood et al. (2017) in observing no significant differences toward a spatial slope of 1.25 in discrimination sensitivity between gray scale and thresholded movie types. Additionally, the peak temporal sensitivity for each movie type was observed in response to stimuli with the intermediate temporal slope of 1.25. Our data agree with the results of Isherwood et al. (2021) and Baker and Graf (2009) with the present study replicating their findings of optimized sensitivity toward gray scale stimuli exhibiting the most natural temporal 1/f^α^ spectral characteristics. Furthermore, the present study has extended these findings by observing that discrimination sensitivity remained optimized in response to dynamic thresholded stimuli, in agreement with the assertion that the photometric properties of a stimulus appear less relevant to the visual system than the fractal characteristics in the context of facilitating sensitivity. This conclusion is based on the continued presence of peak sensitivity toward dynamic stimuli exhibiting natural fractal structure yet highly altered 1/f^α^ spectra.

Our findings lead us to question why the human visual system seems to be tuned systematically toward the structure of natural scenes in both space and time. We suggest that the modulating role of fractal structure may be due to the influence of the environment as a biological constraint throughout the development of human sensory and perceptual systems (Barlow, 1961; Simoncelli and Olshausen, 2001). Examining the physiological consequences of restricting visual experience in cats during early visual developmental has found that neural activation and arrangement significantly differed to controls (Hirsch and Spinelli, 1971; Stryker et al., 1978). Although the feline visual system differs in several respects from the human visual system, it seems that human visual system development proceeds in a similar manner (Movshon & Van Sluyters, 1981). As such, an examination of the coding properties inherent in natural environmental motion provides key insights into the factors constraining and guiding visual processes as they occur in time. Because natural scenes contain statistical regularities, the probability of a particular signal being presented is higher for certain properties in comparison with others. To exploit this redundancy, the visual system may, therefore, have allocated more resources toward these properties to optimize the processing of visual input, such as the 1/f^α^ amplitude spectrum (Barlow, 1961; Knill et al., 1990). Natural scene structure may be statistically more efficient to process when compared with natural illumination conditions in space and time. Scene illumination can be influenced by factors such as time of day (e.g., sunrise vs. sunset) and weather conditions (e.g., cloudy vs. sunny), whereas the fractal microstructure and macrostructure contained in a natural scene is less susceptible to such fluctuations. We propose that the fractal geometry of natural scenes may be a more reliable statistical property in the ambient optic array than luminance-based properties. If the visual system has evolved to process natural scenes as efficiently as possible, our findings may provide psychophysical support for theories of efficient encoding. Because the brain itself exhibits fractal-like neural structures embedded across a multitude of spatial scales, it is fitting that this complex sensory organ seems to be optimized toward myriad fractal structures in the external world (Mac Cormac & Stamenov, 1996; Gibson, 1979; Taylor & Spehar, 2016).

We also observed that sensitivity was increased when the spatial slope and temporal slope were concordant in space and time, corroborating the findings of Billock et al. (2001a) and Isherwood et al. (2021). Stimuli with the shallowest spatial slope (0.25) elicited peak sensitivity at the shallow temporal slope condition of 0.75. Sensitivity for stimuli with a steep spatial slope (2.25) was elevated at the steeper temporal slope conditions (1.75 and 2.25), and approached the sensitivity of responses for the most natural stimuli in the set (spatial slope of 1.25 and temporal slope of 1.25). Although only speculative at this point, increased sensitivity for stimuli congruent in space and time may be meaningful when considering real-world natural scenes; whether animate or inanimate, smaller objects such as ants swarming or grass stalks rippling in the wind typically display faster motion than larger objects, such as elephants walking or large tree boughs swaying. The spatiotemporal tuning evident in our data may indicate a systematic sensitivity toward congruent spatiotemporal patterns characteristic of dynamic natural objects or events. This conclusion may be considered reasonable given the adaptive nature of neural system development based on environmental conditions (Simoncelli & Olshausen, 2001).

## Conclusions

Our results show that the fractal structure within natural motion is vital in facilitating effective discrimination. If the visual system is guided by efficient encoding principles and has developed to exploit the most predictable properties in the environment it depends on (Barlow, 1961), the self-affine fractal structure in dynamic natural scenes is put forward as a more reliable environmental signal than luminance-based scene information. Further investigation into this systematic tuning would benefit from the use of stimuli with greater structural and luminance-based differences, such as spatiotemporal 1/f^α^ noise variations with extracted boundary contours. Nguyen and Spehar (2021) used static edges-only stimuli to measure discrimination sensitivity toward fractal structure in the spatial domain. Such edge stimuli display the contours extracted from a set of thresholded images. This process further alters the 1/f^α^ spectral properties of thresholded stimuli in comparison to a parent gray scale set, yet retains similar structural characteristics (when measured by fractal *D*). The authors observed peak sensitivity across gray scale, thresholded, and edge stimuli toward the most natural spatial slope in the set: 1.25 (Nguyen & Spehar, 2021). Including a dynamic edges-only stimulus set in future work would aim to replicate these findings observed in the spatial domain, and extend our examination into the visual system's preferential, spatiotemporal tuning toward natural fractal structure. Furthermore, our research would benefit from an empirical investigation into the dynamic fractal structure evident in various types of natural motion. To our current knowledge, no comprehensive analysis of temporal fractal *D* values exists for dynamic natural scenes. This goal may be achieved through analyses of natural movie databases or documentaries. Quantifying these statistics would provide researchers with a greater body of data for comparing human visual sensitivity with the regularities inherent in real-world natural motion. At present, our findings provide support for a model of sensory processing in the visual system modulated by the fractal structure of dynamic natural scenes.

## Supplementary Material

Supplement 1
