# Supplementary material for: Nature in motion: The tuning of the visual system to the spatiotemporal properties of natural scenes
Source: J Vis. 2022 May 19;22(6):7. doi: 10.1167/jov.22.6.7 (PMC9123491; doi:10.1167/jov.22.6.7)
Supplement: Supplement 1 [file jovi-22-6-7_s001.pdf]

Supplementary Materials

Table S-1. Spatial Measurements: Measured 1/f$^{\alpha}$ slope (Spatial Slope) and spatial fractal D values across Greyscale and Thresholded movie types in the spatial domain

		Movie Type		Measured Difference
	Spatial Slope (input)	Greyscale	Thresholded	Greyscale and Thresholded
Spatial Slope (measured)	0.25	0.26 (< 0.01)	0.20 (< 0.01)	0.06
	1.25	1.27 (0.01)	1.10 (0.01)	0.17
	2.25	2.37 (0.11)	1.51 (0.02)	0.85
Spatial Fractal *D* (measured)	0.25	1.97 (< 0.01)	1.97 (< 0.01)	0.00
	1.25	1.65 (0.01)	1.65 (0.01)	0.00
	2.25	1.00 (0.02)	0.99 (0.02)	0.01

Notes: Reported Spatial Slope values have been averaged across the 640 frames presented in each Temporal Slope condition (128 frames x 5 Temporal Slope). Values in parentheses indicate standard deviation. Results have been rounded to two decimal places.

Spatial Measurements

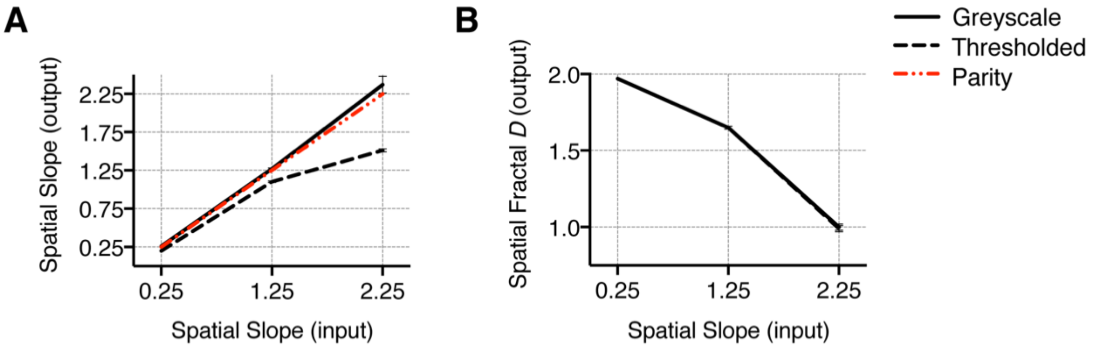

Figure S-1. Measurements in the spatial domain: Measured output 1/f$^{\alpha}$ slope and fractal D values after 8-bit conversion. ***A)*** *Output Spatial Slope plotted as a function of input Spatial Slope and displayed against a line of parity (indicating identical input/output values). Averaged across Temporal Slope conditions, output Spatial Slope conditions can be seen to deviate markedly between movie types, particularly at Spatial Slope 2.25.* ***B)*** *Spatial fractal D output measured for each movie type plotted as a function of Spatial Slope.* ***C)*** *There is comparatively minimal variance in fractal D compared with the range of measured output for α, particularly at Spatial Slope 2.25. This illustrates the high degree of structural similarity retained despite the vast photometric changes to the stimuli. Error bars indicate the standard error of the mean.*

Table S-2. Temporal Measurements: Measured Temporal Slope and temporal fractal D values across Greyscale and Thresholded movie types in the temporal domain after 8-bit conversion

	Temporal Slope (input)	Movie type		Measured difference
		Greyscale	Thresholded	Greyscale and Thresholded
Temporal Slope (measured)	0.25	0.21 (0.07)	0.08 (0.07)	0.12
	0.75	0.60 (0.24)	0.27 (0.22)	0.32
	1.25	0.92 (0.51)	0.51 (0.31)	0.41
	1.75	1.30 (0.64)	0.68 (0.35)	0.62
	2.25	1.59 (0.07)	0.73 (0.42)	0.87
Temporal Fractal *D* (measured)	0.25	1.69 (0.01)	1.79 (0.02)	0.10
	0.75	1.50 (< 0.01)	1.58 (0.01)	0.07
	1.25	1.29 (0.01)	1.33 (< 0.01)	0.03
	1.75	1.19 (0.01)	1.22 (0.02)	0.02
	2.25	1.17 (< 0.01)	1.19 (0.02)	0.02

Notes: Reported Temporal Slope values have been averaged across the three Spatial Slope conditions (0.25, 1.25, 2.25). Values in parentheses indicate standard deviation. Results have been rounded to two decimal places.

Temporal Measurements

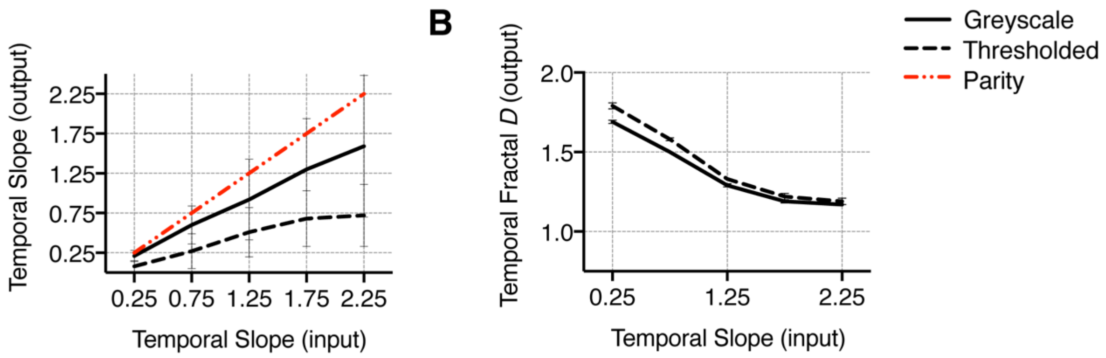

Figure S-2. Measurements in the Temporal Domain: output 1/f$^{\alpha}$ slope and fractal D values after 8-bit conversion. ***A)*** *Output Temporal Slope averaged across Spatial Slope, plotted as a function of input Temporal Slope and displayed against a line of parity (no difference). Similar to the measured output slopes in the spatial domain, output Temporal Slope conditions are notably different between movie types.* ***B)*** *Output temporal fractal D values averaged across Spatial Slope for each movie type and plotted as a function of Temporal Slope.* ***C)*** *Plotting the measured difference for each parameter reveals substantially less difference in measured D between movie types when compared to the range of the difference in measured α, which is particularly large at the higher Temporal Slope conditions. Error bars indicate the standard error of the mean.*

Table S-3. Summary of measured spatial slope and spatial fractal D estimates across all stimulus conditions after 8-bit conversion.

	Temporal Slope (input)	Spatial Slope (measured)		Spatial Fractal *D* (measured)	
		Greyscale	Thresholded	Greyscale	Thresholded
0.25 (input)	0.25	0.26 (0.02)	0.20 (0.02)	1.97 (< 0.01)	1.97 (< 0.01)
	0.75	0.26 (0.02)	0.20 (0.02)	1.97 (< 0.01)	1.97 (< 0.01)
	1.25	0.26 (0.01)	0.20 (0.02)	1.97 (< 0.01)	1.97 (< 0.01)
	1.75	0.27 (0.01)	0.20 (0.02)	1.97 (< 0.01)	1.97 (< 0.01)
	2.25	0.27 (0.01)	0.20 (0.02)	1.97 (< 0.01)	1.97 (< 0.01)
1.25 (input)	0.25	1.26 (0.13)	1.09 (0.09)	1.66 (0.03)	1.66 (0.03)
	0.75	1.28 (0.11)	1.10 (0.09)	1.66 (0.04)	1.66 (0.04)
	1.25	1.26 (0.10)	1.11 (0.10)	1.65 (0.04)	1.65 (0.04)
	1.75	1.28 (0.09)	1.12 (0.10)	1.64 (0.03)	1.64 (0.03)
	2.25	1.28 (0.07)	1.11 (0.10)	1.64 (0.03)	1.64 (0.03)
2.25 (input)	0.25	2.22 (0.28)	1.49 (0.09)	1.02 (0.05)	1.01 (0.05)
	0.75	2.28 (0.27)	1.50 (0.10)	1.01 (0.06)	1.00 (0.05)
	1.25	2.40 (0.34)	1.53 (0.09)	0.99 (0.04)	0.98 (0.04)
	1.75	2.45 (0.30)	1.53 (0.07)	0.99 (0.04)	0.98 (0.03)
	2.25	2.49 (0.21)	1.53 (0.06)	0.98 (0.03)	0.97 (0.03)

Notes: Reported Spatial Slope values have been averaged across the 128 frames in condition. Values in parentheses indicate standard deviation in estimated spatial slope or spatial fractal D across frames. Results have been rounded to two decimal places.

Table S-4. Summary of measured temporal slope and temporal fractal D estimates across all stimulus conditions after 8-bit conversion.

	Temporal Slope (input)	Temporal Slope (measured)		Temporal Fractal *D* (measured)	
		Greyscale	Thresholded	Greyscale	Thresholded
0.25 (input)	0.25	0.25	0.16	1.68 (0.02)	1.80 (0.03)
	0.75	0.75	0.49	1.50 (0.03)	1.57 (0.09)
	1.25	1.24	0.77	1.30 (0.05)	1.33 (0.10)
	1.75	1.72	0.95	1.20 (0.05)	1.24 (0.06)
	2.25	2.19	1.01	1.17 (0.04)	1.21 (0.04)
1.25 (input)	0.25	0.24	0.07	1.68 (0.02)	1.80 (0.03)
	0.75	0.72	0.27	1.50 (0.04)	1.58 (0.09)
	1.25	1.19	0.60	1.30 (0.06)	1.33 (0.11)
	1.75	1.61	0.81	1.20 (0.05)	1.21 (0.07)
	2.25	2.01	0.88	1.17 (0.04)	1.17 (0.04)
2.25 (input)	0.25	0.13	0.02	1.69 (0.02)	1.77 (0.03)
	0.75	0.32	0.06	1.50 (0.04)	1.58 (0.07)
	1.25	0.33	0.16	1.29 (0.06)	1.32 (0.08)
	1.75	0.56	0.28	1.19 (0.04)	1.20 (0.05)
	2.25	0.56	0.28	1.17 (0.03)	1.19 (0.04)

*Notes: Reported temporal fractal D values have been averaged across all possible pixel coordinates (256*256 = 65,536). Values in parentheses indicate standard deviation in estimated temporal fractal D across pixel coordinates. Results have been rounded to two decimal places.*

Table S-5. Summary of measured spatial slope and spatial fractal D estimates across all stimulus conditions prior to 8-bit conversion.

	Temporal Slope (input)	Spatial Slope (measured)		Spatial Fractal *D* (measured)	
		Greyscale	Thresholded	Greyscale	Thresholded
0.25 (input)	0.25	0.24 (0.01)	0.02 (0.01)	1.97 (< .01)	1.97 (< 0.01)
	0.75	0.24 (0.01)	0.02 (0.01)	1.97 (< 0.01)	1.97 (< 0.01)
	1.25	0.24 (0.01)	0.02 (0.01)	1.97 (< 0.01)	1.97 (< 0.01)
	1.75	0.24 (0.01)	0.02 (0.02)	1.97 (< 0.01)	1.97 (< 0.01)
	2.25	0.24 (0.01)	0.02 (0.01)	1.97 (< 0.01)	1.97 (< 0.01)
1.25 (input)	0.25	1.23 (0.01)	0.55 (0.01)	1.66 (0.03)	1.66 (0.03)
	0.75	1.23 (0.01)	0.55 (0.02)	1.66 (0.04)	1.66 (0.04)
	1.25	1.23 (0.01)	0.55 (0.01)	1.65 (0.04)	1.65 (0.04)
	1.75	1.23 (0.01)	0.55 (0.01)	1.64 (0.03)	1.64 (0.03)
	2.25	1.23 (0.01)	0.55 (0.03)	1.64 (0.03)	1.64 (0.03)
2.25 (input)	0.25	2.23 (0.01)	1.09 (0.03)	1.02 (0.05)	1.01 (0.05)
	0.75	2.23 (0.01)	1.09 (0.03)	1.01 (0.06)	1.00 (0.05)
	1.25	2.23 (0.01)	1.09 (0.03)	0.99 (0.04)	0.98 (0.04)
	1.75	2.23 (0.02)	1.10 (0.03)	0.99 (0.04)	0.98 (0.03)
	2.25	2.23 (0.02)	1.11 (0.02)	0.98 (0.03)	0.97 (0.03)

Notes: Reported Spatial Slope values have been averaged across the 128 frames in condition. Values in parentheses indicate standard deviation in estimated spatial slope or spatial fractal D across frames. Results have been rounded to two decimal places.

Table S-6. Summary of measured temporal slope and temporal fractal D estimates across all stimulus conditions prior to 8-bit conversion.

	Temporal Slope (input)	Temporal Slope (measured)		Temporal Fractal *D* (measured)	
		Greyscale	Thresholded	Greyscale	Thresholded
0.25 (input)	0.25	0.25	0.15	1.68 (0.02)	1.80 (0.03)
	0.75	0.75	0.45	1.50 (0.03)	1.57 (0.09)
	1.25	1.25	0.71	1.30 (0.05)	1.33 (0.10)
	1.75	1.75	0.85	1.20 (0.05)	1.24 (0.06)
	2.25	2.25	0.87	1.17 (0.04)	1.21 (0.04)
1.25 (input)	0.25	0.25	0.02	1.68 (0.02)	1.80 (0.03)
	0.75	0.74	0.17	1.50 (0.04)	1.58 (0.09)
	1.25	1.25	0.48	1.30 (0.06)	1.33 (0.11)
	1.75	1.75	0.69	1.20 (0.05)	1.21 (0.07)
	2.25	2.24	0.74	1.17 (0.04)	1.17 (0.04)
2.25 (input)	0.25	0.23	0.00	1.69 (0.02)	1.77 (0.03)
	0.75	0.72	0.01	1.50 (0.04)	1.58 (0.07)
	1.25	1.23	0.06	1.29 (0.06)	1.32 (0.08)
	1.75	1.75	0.17	1.19 (0.04)	1.20 (0.05)
	2.25	2.29	0.22	1.17 (0.03)	1.19 (0.04)

*Notes: Reported temporal fractal D values have been averaged across all possible pixel coordinates (256*256 = 65,536). Values in parentheses indicate standard deviation in estimated temporal fractal D across pixel coordinates. Results have been rounded to two decimal places.*

Temporal Slope measurement of movie stimulus: GS 2.25 TS 2.25

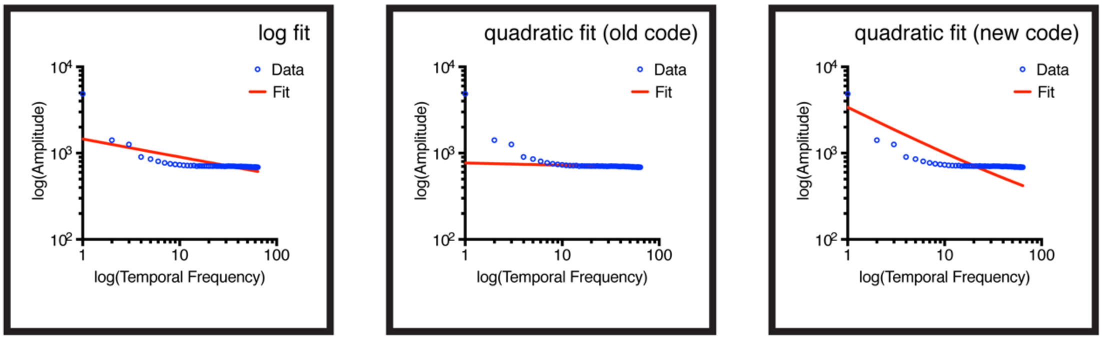

temporal slope = 0.21
R2 (log space, all points) = 0.50

temporal slope = 0.03
R2 (log space, cut off: 9) = 0.85
R2 (log space, incl. all points) = 0.01
R2 (linear space, cut off: 9) = 0.85
R2 (linear space, incl. all points) = 0.01

temporal slope = 0.56
R2 (log space, all points) = 0.11
R2 (linear space, all points) = 0.62

Figure S-3. Demonstration of how estimating the slope of the $1/f^{\alpha}$ amplitude spectrum depends on the fitting method (particularly after 8-bit conversion). The example stimulus used in this demonstration was our Greyscale Spatial Slope 2.25 Temporal Slope 2.25 condition, and specifically we measure the $1/f^{\alpha}$ temporal amplitude spectrum of this stimulus. ***Left panel****: a depiction of the traditional method of estimating the slope: fitting a linear function to the $1/f^{\alpha}$ amplitude spectrum in log-log space.* ***Middle panel****: a depiction the method used in Isherwood et al. (2021), where a quadratic function was fitted to the amplitude spectrum in linear space, with the results of the fit depicted on a log-log axis. Due to systematic deviations from linearity across low temporal frequencies, Isherwood et al. (2021) used an Akaike Information Criterion to compare quadratic fits in linear space and linear fits in log-log space across a chosen range of points to be cut off from the $1/f^{\alpha}$ amplitude spectrum (the first 10 points). While this method yielded accurate measures in Isherwood et al. (2021), it yielded poor fits (R2) for the stimuli used in the present study.* ***Right panel****: a depiction of the new fitting method used in the present study. Here we choose a range of values to cut off that is more flexible across any movie size (the bottom 2% of points across frames). We also instead only compare the quadratic fit (R2) when each point is removed rather than using an AIC to compare linear space and log-log space fits. As shown in the plot of the fit, this yielded a better estimate of the $1/f^{\alpha}$ amplitude spectrum slope that is not biased by the rounding of high frequencies from double to integer space (e.g. 8-bit conversion) that is magnified on a log-log axis.*

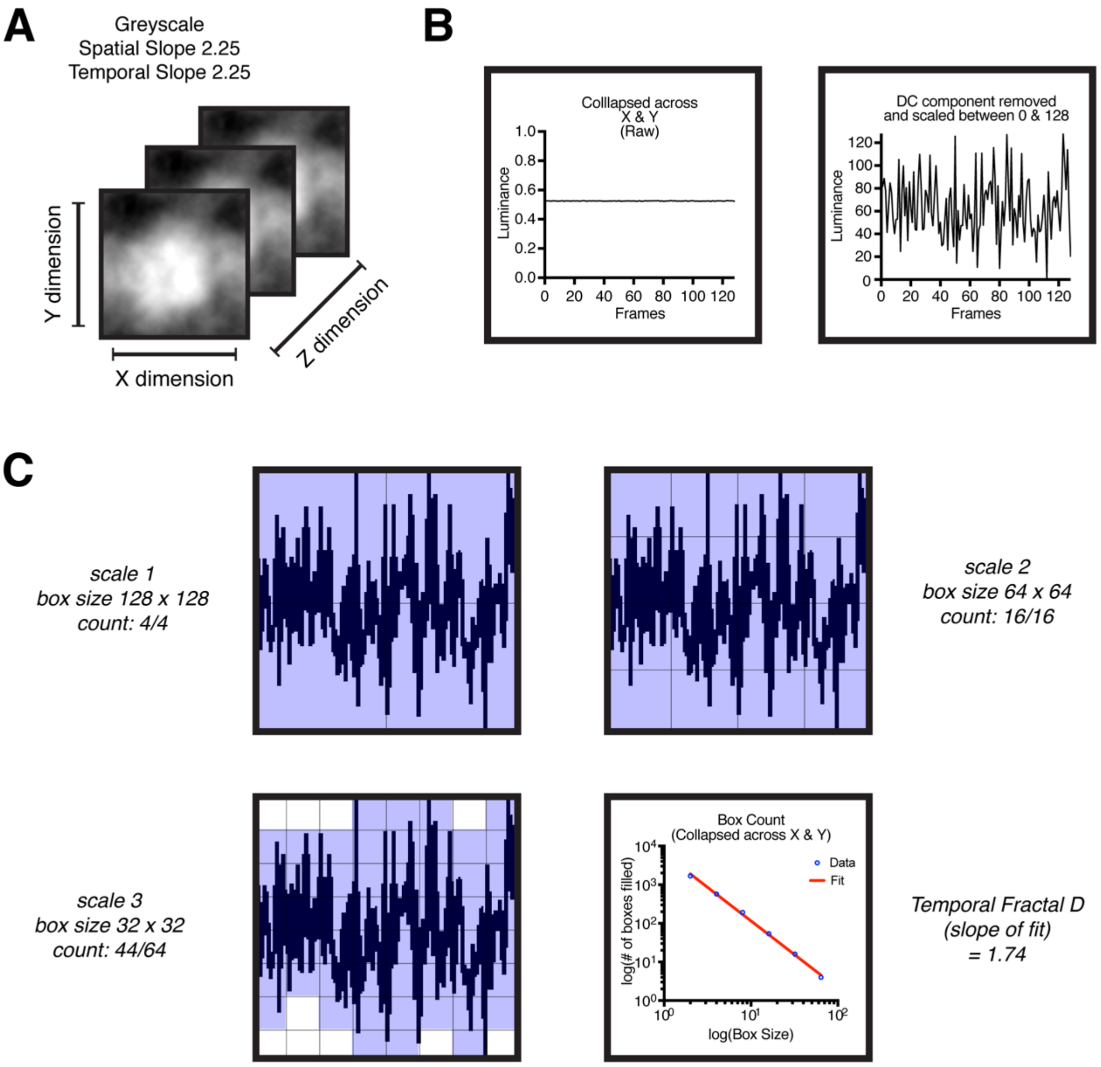

*Figure S-4. Demonstration of measuring temporal fractal D when averaging across spatial dimensions (X & Y). **A)** The stimulus condition used in this demonstration was Greyscale Spatial Slope 2.25 Temporal Slope 2.25. **B)** Before box-counting, the luminance values of the stimulus were averaged across spatial dimensions (X & Y). The left plot depicts averaged luminance over frames (Z dimension). Note how little variance in luminance there is across frames. The right plot depicts the same data plotted on the left, but with the DC component removed and with values scaled between 0 & 128. Note how there now appears to be a large amount of luminance variance across frames. **C)** A depiction of the box counting procedure, which was conducted on the plot depicted on the right panel of B. The temporal fractal D estimate was 1.74. This value is artificially inflated since plotting luminance across frames with the DC component removed increases luminance variance over time. See Figure 4 to compare this method with measuring temporal fractal D across each pixel coordinate separately, which instead yielded an estimate of 1.17.*

Table S-7. *Summary of Bonferroni-corrected pairwise comparisons following the significant main effect of Spatial Slope.*

Spatial Slope *(i)*	Spatial Slope *(j)*	Mean difference *(i - j)*	Standard Error	*p*	95% CI	
					Lower Bound	Upper Bound
0.25	1.25	- .447*	.024	< .001	- .495	- .399
	2.25	- .346*	.026		- .399	- .294
1.25	0.25	.447*	.024	< .001	.399	.495
	2.25	.101*	.026		.048	.154
2.25	0.25	.346*	.024	< .001	.294	.399
	1.25	- .101*	.026		- .154	- .048

Note. CI = confidence interval for the mean difference. *The mean difference is significant at the .05 level.

Table S-8. *Summary of Bonferroni-corrected pairwise comparisons following the significant main effect of Temporal Slope.*

Temporal Slope *(i)*	S *(j)*	Mean difference *(i - j)*	Standard Error	*p*	95% CI	
					Lower Bound	Upper Bound
0.25	0.75	- .406*	.021	< .001	- .477	- .365
	1.25	- .778*	.040	< .001	- .856	- .699
	1.75	- .424*	.010	< .001	- .444	- .404
	2.25	- .273*	.010	< .001	- .292	- .254
0.75	0.25	.406*	.021	< .001	.365	.447
	1.25	- .372*	.033	< .001	- .438	- .306
	1.75	- .018	.021	.377	- .059	.023
	2.25	.133*	.021	< .001	.092	.174
1.25	0.25	.778*	.040	< .001	.699	.438
	0.75	.372*	.033	< .001	.306	.427
	1.75	.354*	.037	< .001	.280	.427
	2.25	.505*	.039	< .001	.428	.582
1.75	0.25	.424*	.010	< .001	.404	.444
	0.75	.018	.021	.377	- .023	.059
	1.25	- .354*	.037	< .001	- .427	- .280
	2.25	.151*	.013	< .001	.126	.176
2.25	0.25	.273*	.010	< .001	.254	.292
	0.75	- .133*	.021	< .001	- .174	- .092
	1.25	- .505*	.039	< .001	- .582	- .428
	1.75	- .151*	.013	< .001	- .176	- .126

Note. CI = confidence interval for the mean difference. *The mean difference is significant at the .05 level.
